# Kuijieyuan Decoction Improved Intestinal Barrier Injury of Ulcerative Colitis by Affecting TLR4-Dependent PI3K/AKT/NF-κB Oxidative and Inflammatory Signaling and Gut Microbiota

**DOI:** 10.3389/fphar.2020.01036

**Published:** 2020-07-29

**Authors:** Baohai Liu, Xuehua Piao, Wei Niu, Qingyu Zhang, Chi Ma, Tong Wu, QiChang Gu, Tingfang Cui, Shuangdi Li

**Affiliations:** ^1^ Department of Gastroenterology, The First Affiliated Hospital, Jinzhou Medical University, Jinzhou, China; ^2^ Department of Traditional Chinese Medicine, The First Affiliated Hospital, Jinzhou Medical University, Jinzhou, China; ^3^ Heart Disease Center, The Affiliated Hospital of Changchun University of Traditional Chinese Medicine, Changchun, China

**Keywords:** TLR4-dependent PI3K/AKT/NF-κB signaling, Kuijieyuan decoction, antioxidant, anti-inflammatory, gut microbiota, ulcerative colitis

## Abstract

**Ethnopharmacological Relevance:**

In Traditional Chinese medicine (TCM) theory, ulcerative colitis (UC) is associated with damp-heat, blood stasis, and intestinal vascular ischemia. Kuijieyuan decoction (KD) is a traditional Chinese medicine based on the above theory and used clinically to alleviate UC injury.

**Methods:**

The main components of KD were analyzed by using high-pressure liquid chromatography (HPLC) and confirmed by UPLC-MS/MS. A UC model was established in rats by using dextran sulfate sodium (DSS) and dead rats (caused by DSS) were excluded from the study. Forty-eight rats were divided into 6 groups, health control (CG), UC model (UG), sulfasalazine (SG), low-dose KD (LG), middle-dose KD (MG), and high-dose KD (HG) groups. UC damage was assessed by hematoxylin and eosin staining and scan electron microscopy. We measured Toll-like receptor 4 (TLR4), p-phosphatidylinositol 3-kinase (PI3K), PI3K, p-Protein kinase B (AKT), AKT, p-nuclear factor kappa B (NF-κB), NF-κB, oxidative stress marker (superoxidase dismutase (SOD), catalase (CAT), glutathione peroxidases (GPx), and malondialdehyde) and inflammatory markers (tumor necrosis factor α (TNFα), interleukin (IL)-1, IL-6 and IL-10) in UC tissues. Gut microbiota was analyzed through16S rRNA sequencing.

**Results:**

The main components of KD consist of gallic acid, paeoniflorin, emodin, berberine, coptisine, palmatine, jatrorrhizine, baicalein and baicalin. The UC model was successfully established by causing intestinal barrier injury with the loss of intestinal villi and destructed mitochondria of intestinal epithelial cells. Both sulfasalazine and KD treatment repaired UC injury, reduced the levels of malondialdehyde, TNFα, IL-1, IL-6, TLR4, p-PI3K, p-AKT, and p-NF-κB, and increased the levels of SOD, GPx, CAT, and IL-10. KD showed a protective function for the UC model in a dose-dependent way. The serum levels of paeoniflorin and baicalin had a strong relationship with the levels of inflammatory and oxidative stress biomarkers. KD treatment increased the proportion of Alloprevotella, Treponema, Prevotellaceae, and Prevotella, and reduced the proportion of Escherichia_Shigella and Desulfovibrio in gut microbiota.

**Conclusions:**

KD improved intestinal barrier injury of ulcerative colitis, antioxidant and anti-inflammatory properties by affecting TLR4-dependent PI3K/AKT/NF-κB signaling possibly through the combination of its main compounds, and improving gut microbiota.

## Introduction

Ulcerative colitis (UC) is a chronic inflammatory disease of the colon caused by unbalanced immune responses to host intestinal microbiota and main threatens to public health (Roselli and Finamore, 2019). Although much progress has been made in diagnosis (Yang et al., 2019; Vadstrup et al., 2020) and therapy (Karjalainen et al., 2020; Kunovszki et al., 2020) for UC in recent years, an effective therapeutic drug is still difficult to be available. Gut microbiota disturbance (Noor et al., 2010) and intestinal barrier injury (Chen et al., 2019) is often associated with a UC risk. In Traditional Chinese medicine (TCM) theory, UC is associated with damp-heat, blood stasis, and intestinal vascular ischemia. According to the concepts, UC should be treated by clearing away heat, eliminating dampness, and warming Yang.

Kuijieyuan decoction (KD) is a prescription traditional Chinese medicine by compiling Chinese philosophical texts based on the above theory and used clinically to alleviate the symptoms associated with UC. KD is made of many vegetables and herbal medicines (Xu et al., 2016) (Supporting File 1) and has long been used for UC therapy (Xu et al., 2016; Yu et al., 2017). Kuijieyuan decoction (KD) consists of *Astragalus mongholicus* Bunge, *Hedyotis diffusa* Willd., *Cirsium undulatumn* (Nutt.) Spreng, *Cirsium setosum* (Willd.) M. Bieb., *Pulsatilla vulgaris* Mill, *Prunella vulgaris* L. subsp. Vulgaris, *Coptis chinensis* Franch, *Polygonum cuspidatum* Sieb. et Zucc., *Atractylodes lancea* (Thunb) DC and *Glycyrrhiza glabra* L. Most of these herbs have antioxidant [such as *A. mongholicus* Bunge (Yu et al., 2005) and *H. diffusa* Willd. (Lu et al., 2000)], anti-inflammatory [such as *C. chinensis* Franch (Aroonrerk and Kamkaen, 2009) and *P. cuspidatum* Sieb. Et Zucc. (Bralley et al., 2008)[ and anti-bacterial [such as *G. glabra* L. (Nirmala and Selvaraj, 2011)] properties. Thus, KD may exert its function through multiple anti-inflammatory, antioxidant and antibacterial properties. However, the effects of KD on gut microbiota and intestinal barrier injury, and the functional molecular mechanism remains widely unknown.

Oxidative stress and inflammatory responses are the main pathogenic factors associated with UC. Therefore, the improvement of antioxidant and anti-inflammatory capacities has become the research hotspot in the prevention of UC progression (Wang et al., 2019; Gerges et al., 2020; Jeon et al., 2020). Baicalin is one of the dominant ingredients of KD and has a specific antioxidant and anti-inflammatory function on UC by inhibiting the changes in p-IKBα/IKBα content (Shen et al., 2019). Baicalin showed protective effects against ischemia/reperfusion liver injury in a rat model by improving antioxidant abilities (Kim et al., 2010). Baicalin exerted anti-inflammatory function by suppressing the production of tumor necrosis factor-α (TNFα) and interleukin-6 (IL-6), and the activation of nuclear factor-κB (NF-κB) or extracellular regulated kinases (Lee et al., 2015).

Paeoniflorin, a monoterpene glycoside, is another important ingredient of KD, and has been reported to display antioxidant and anti-inflammatory properties in the UC therapy (Shen et al., 2019). Paeoniflorin protected against alpha-naphthylisothicaynate-induced cholestasis in a rat model by reducing oxidative stress (Zhao et al., 2013). Paeoniflorin has anti-inflammatory properties by inhibiting NF-κB expression in chronic hypoperfusion rats (Tang et al., 2010). Therefore, the antioxidant and anti-inflammatory function may be underlying pharmacological mechanisms of KD in UC therapy.

The Toll-like receptor 4 (TLR4)-dependent phosphatidylinositol 3-kinase (PI3K)/Protein kinase B (AKT)/NF-κB signaling is closely associated with the oxidative stress and inflammatory responses (Jiang et al., 2018; Thummayot et al., 2018). However, whether KD exerts its anti-inflammatory and antioxidant effects on TLR4-dependent PI3K/AKT/NF-κB signaling in the UC therapy remains unknown. Therefore, in this study, we aimed to explore the related molecular mechanisms for the protective effects of KD in the UC model.

## Materials and Methods

### Chemicals

Purified baicalin (>95%, CAS No.: 21967-41-9), paeoniflorin (>98%, CAS No.: 23180-57-6), gallic acid (>98%, CAS No. 149-91-7), berberine (>98%, CAS No.: 633-65-8), coptisine (>98%, CAS No.: 6020-18-4), palmatine (>98%, CAS No.: 10605-02-4), jatrorrhizine (>98%, CAS No.: 222-817-3) and baicalein (>98%, CAS No.: 491-67-8) were purchased from Sigma (St. Louis, MO, USA). All chemicals were dissolved in sterile saline to the concentration of 10 mg/ml. Dextran sulfate sodium (DSS, Cat. No. 45-42867-5G) was purchased from Sigma and dissolved in 0.9% NaCl to final 1 mg/ml. KD was purchased from Tianjin Tasly Pharmaceutical Co., Ltd. (batch number 60060101, Tianjin, China) and prepared as follows (Voucher number): 30 g of *A. mongholicus* Bunge (20100478, **Figure S1**), 40 g of *H. diffusa* Willd. (PS1034MT01, **Figure S2**), 10 g of *C. undulatumn* (Nutt.) Spreng (**Figure S3**), 10 g of *C. setosum* Willd. M. Bieb (PS0611MT04, **Figure S4**), 40 g of *P. vulgaris* Mill (**Figure S5**), 10 g of *P. vulgaris* L. subsp. Vulgaris (MIB: ZPL:03560, **Figure S6**), 10 g of *C. chinensis* Franch (PS0915MT01, **Figure S7**), 15 g of *P. cuspidatum* Siebold & Zucc. (**Figure S8**), 15 g of *A. lancea* (Thunb) DC (SKP 051011201, **Figure S9**), and 10 g of *G. glabra* L.) (8600100, **Figure S10** **)**. The herbs were rinsed and soaked in 2-liter cold water for 30 min, and then boiled in water under reflux for 2 h. After the concentration and filtration, the medicinal solution was concentrated to 1.0 g/ml of crude drug, and stored in a refrigerator at 4°C. The required medicinal recipe was provided by the Chinese Medicine Bureau of the First Affiliated Hospital of Jinzhou Medical University.

### HPLC Analysis of KD and Rat Serum

The main ingredients in KD were detected by using a Shimadzu high pressure liquid chromatography (HPLC, Kyoto, Japan). The standards (0.002 g gallic acid, 0.002 g Paeoniflorin, 0.005 g baicalin, 0.003 g berberine, 0.004 g coptisine, 0.003 g palmatine, 0.004 g jatrorrhizine, 0.005 g baicalein and 0.005 g sulfasalazine) were placed in the same 20 ml of flask, dissolved in methanol and diluted to 0.01, 0.02 and 0.02 mg/ml, respectively. Two-gram KD was placed into a mortar and ground into fine powder, and placed in a 30 m flask. Thirty milliters of methanol was added and sonicated for 1 h. The dominant ingredients of KD were subsequently eluted by using the following chromatographic conditions: Column, Amethyst C18-H (4.6 mm × 250 mm, 5 µm, 120 Å); mobile phase: acetonitrile (A) −0.4% phosphoric acid aqueous solution (B), gradient elution (0–10 min, 4% A; 10–20 min, 4% A → 16% A; 20–35 min, 16% A → 55% A; Flow rate: 1.0 ml/min; Detection wavelength: 250 nm; Column temperature: 25 °C and Injection volume: 10 μl. Rat serum was also analyzed using the same condition to investigate the levels of sulfasalazine and the main chemicals of KD in the rats.

### Ultra-Performance Liquid Chromatography-Tandem Mass Spectrometer (UPLC-MS/MS) Analysis of KD Extract

The UPLC-MS/MS analysis was performed using UPLC/MS/MS ACQUITY Xevo TQ from Waters (Milford,.MA, USA). The following UPLC condition was used: The chromatographic column a Fortis Xi C18 column (50 mm×2.1 mm, 1.7 μm); the mobile phase acetonitrile (A) −0.1% formic acid aqueous solution (B), gradient elution (0–1 min, 10%A; 1–4 min, 10% → 45%A; 4–7 min, 45% → 60%A; 7–8 min, 60%A; 8–8.5 min, 60% → 10%A); Flow rate: 0.3 ml/min; column temperature: 40°C; total running time 10 min; and injection volume 2 μl. The following MS conditions were used: dissolvent gas: nitrogen (600 L/h); capillary voltage: 2.5 kV (positive ion mode); ion source: ESI source; ion source temperature: 150°C and dissolvent temperature: 500°C. Multiple reaction monitoring (MRM) method for quantitative analysis. Multiple reaction monitoring (MRM) transitions m/z 171.1 → 125.0, m/z 399.2 → 367.1, m/z 481.2 → 198.0, m/z 271.2 → 240.1, m/z 337.0 → 320.1, m/z 353.0 → 336.1, m/z 339.0 → 322.1, m/z 321.0 → 292.1, m/z 271.1 → 123.0 and m/z 446.1 → 269.0 were selected for determining gallic acid, sulfasalazine, paeoniflorin, emodin, berberine, palmatine, jatrorrhizine, and coptisine, baicalein and baicalin.

### Establishment of the UC Model

All animal-related procedures were approved by the Institutional Animal Care and Use Committee of Jinzhou Medical University according to international, national and institutional rules considering animal experiments (Jinzhou, China). Sprague–Dawley (SD) rats (8 weeks old; weighing 200–220 g) were purchased from the animal center of Jinzhou Medical University (Jinzhou, China). The rats were housed in a standard cage under light/dark 12:12, with the light on from 6 am to 6 pm, and given a standard laboratory diet containing 23% protein and water *ad libitum*. UC model was induced by using the following protocol. The rats received 5% dextran sodium sulfate (DSS) in distilled water for 5 d, and changed to tap water for 10 d. The whole process was repeated three times and a chronic UC model was established. The general condition, body weight, stool characteristics, and occult blood or gross bloody stool of the rats were observed daily. The mortality rate reached up to 30% after three times of DSS treatments. The dead rats were excluded from the present study. After the modeling was completed, two rats in the normal group and the model group were sacrificed. The histopathological scores of the two groups of rats were compared and comprehensively analyzed to determine whether the model was successfully induced.

### Animal Grouping

After 7-day model establishment, all rats administered twice daily by gavage and divided into 6 groups and treated orally, the vehicle (CG, 3-ml saline solution daily), UC model (UG, 3-ml saline solution daily), sulfasalazine [SG, 5 mg/kg sulfasalazine daily (Soliman et al., 2019)], low-dose KD (LG, 1 ml/kg daily), middle-dose KD (MG, 2 ml/kg) and high-dose KD (HG, 10 ml/kg) groups. After 15-day gavage, all rats were sacrificed.

### Measurement of Biochemical Indexes in Colon Tissues

Ten milligrams of colon tissue was ground in liquid nitrogen and lysed by using RIPA lysis (CST, Danvers, MA, USA). The supernatant was prepared *via* centrifugation at 12,000×*g* for 10 min and stored at −20°C for ELISA measurement. The levels of tumor necrosis factor α (TNFα) (ab100747), interleukin (IL)-1 β (ab100704), IL-6 (ab100713) and IL-10 (ab100697) were measured by using the ELISA kits from Abcam (San Francisco, CA, USA). The levels of superoxide dismutase (SOD) (#MBS080359) and catalase (CAT) (#MBS775862) and glutathione peroxidase (GSPx) (#MBS049725) were also evaluated using the kits from MyBioSource, Inc. (San Diego, CA, USA). All biochemical indexes were measured on an automatic chemical analyzer (Hitachi, Tokyo, Japan). MDA was measured by using UV–Vis spectroscopy at 267 nm in 10 mM Na_3_PO_4_ buffer pH 11 according to the molar absorbing coefficient 31,500 M^−1^cm^−1^ (Esterbauer et al., 1991). Wavelength scanning of MDA fractions was conducted *via* the wavelength against a control group without 1,3-propanediol and MDA.

### Measurement of Disease Activity Index (DAI) and Colon Length

The clinical assessment of disease severity was evaluated using a scoring procedure every day by combining scores of weight loss, stool consistency and fecal bleeding. DAI is usually calculated as the sum of the body weight loss (scored as: 0, none; 1, 1–5%; 2, 5–10%; 3, 10–20%; 4, over 20%), presence or absence of fecal blood (scored as: 0, negative hemoccult; 2, positive hemoccult; 4, gross bleeding) and stool consistency (scored as: 0, well-formed pellets; 2, loose stools; 4, diarrhea) (Schwanke et al., 2013). On day 15, the rats were sacrificed through intraperitoneal injection of phenobarbital sodium (50 mg/kg) and their colon length was compared among different groups. Colon tissue injuries were detected by following histopathological analysis.

### Scanning Electron Microscopy (SEM) Observation of Intestinal Barrier

For SEM observation, 5 mm^2^ of pieces were cut from each rat after 15-day KD treatment and fixed with 1% osmium tetroxide for 2 h at 4°C. The tissues were rinsed, dehydrated in ethyl alcohol, dried with carbon dioxide, covered with gold, and examined under SEM JSM-6610lv (Jeol, Japan) with an INCA SDD X-MAX energy dispersive micro-analyzer.

### Histological Analysis of Pancreas Tissues

UC or colon tissues were extracted after a 15-day model establishment. UC tissues were fixed in 4% paraformaldehyde and embedded in paraffin and remaining tissues were stored at −80 °C. The embedded pancreatic tissues were cut into 2–3 μm slices and stained with hematoxylin and eosin (H&E). The numbers of inflammatory cell infiltration, bleeding and necrotic cells were calculated. The severity of colon tissue damage was assessed by using pathological score = edema score + necrosis score + inflammatory cellular infiltration score + bleeding score. Five slices were evaluated in each group.

### Immunohistochemistry Analysis

Immunohistochemistry analysis was conducted to evaluate the expression of TLR4, p-PI3K, p-AKT, and p-NF-κB. The paraffin-embedded tissue sections were deparaffinized and treated with hydrogen peroxide (3 m/v) for 15 min to remove endogenous peroxidase. Antigen retrieval was performed by blocking the samples in goat serum for 10 min at 22°C. The following antibodies were added and incubated 12 h at 4°C, including anti-TLR4 antibody (ab13867, 1:500), anti-p-PI3K p85 antibody (ab86714, 1:500), anti-p-AKT antibody (ab38449, 1:500), and or anti-p-NF-κB antibody (ab86299) from Abcam (Cambridge, MA, USA). Anti-PI3K antibody (ab133595), anti-AKT antibody (ab8805), and or anti-NF-κB antibody (ab28849) were also purchased from Abcam and used for the following Western Blot. A biotin-labeled goat anti-rabbit IgG secondary (1:1,000) was added followed by incubation at 37°C for 10 min. The slides were then incubated at 37°C for 10 min with peroxidase-conjugated streptavidin (Sigma, S5512). The sections were stained with 3,3’-diaminobenzidine (DAB, Sigma) and counterstained with hematoxylin (Sigma). The color separation was conducted by using 2% hydrochloride and alcohol, followed by 15-min washing. Each sample was observed in five fields and target protein signals were stained with brown. The positive rates were calculated as the number of positive cells/number of total cells by using an image analyzer (Image-Pro Plus 5.1, MediaCybernetics, MD, USA).

### Reverse Transcription-Quantitative PCR (RT-qPCR)

RNA was extracted from 5-mg colon tissues using TRIzol reagent (TIANGEN, Beijing, China). cDNA was prepared by using a reverse transcription kit (Bioteck, Beijing, China) according to the manufacturer’s instructions. The following primers were used: TLR4 forward primer 5’-CATGGCATTGTTCCTTTCCT-3’ and reverse primer 5’-CATGGAGCCTAATTCCCTGA-3’; PI3K forward primer 5’-TTAAACGCGAAGGCAACGA-3’ and reverse primer 5’-CAGTCTCCTCCTGCTGTCGAT-3’; AKT forward primer 5’-AAAGAGCGCATGAGTGGACG-3’ and reverse primer 5’-CGTGGTCCTCCTTGTAGTAG-3’; NF-κB forward primer 5’-AGAGCAACCGAAACAGAGAGG-3ʹ and reverse primer 5’-TTTGCAGGCCCCACATAGTT-3’ and β-actin forward primer 5’-AAGTCCCTCACCCTCCCAAAAG-3’ and reverse primer 5’-AAGCAATGCTGTCACCTTCCC-3’. qPCR was conducted using SYBR-Green (Invitrogen, USA) (dilution 1:1,000 with deionized water) for 5 min on an Applied Biosystems StepOne Plus real-time PCR machine (Applied Biosystems, Inc., CA, USA). The levels were detected and the relative mRNA levels were normalized to β-actin using the ΔΔCt method.

### Western Blot Analysis

Ten milligram colon tissues were ground in liquid nitrogen and total protein was extracted by using RIPA lysis (CST, Danvers, MA, USA). Protein concentration quantified using the BCA kit (TaKaRa, Dalian, China). HRP-conjugated goat anti-rabbit IgG H&L (ab6721) secondary antibodies were from Abcam (Abcam, San Francisco, CA, USA). The proteins were separated by SDS-PAGE and transferred to the PVDF membrane in the transfer buffer at 100 V for 2–3 h. The membrane was blocked for 1 h at ambient room temperature in 10% non-fat milk, and probed with antibodies against the above primary antibodies for 2 h at 37°C, rinsed four times with PBTB, incubated 2 h at 37°C in secondary antibodies, washed extensively in PBS. Images were acquired on an Odyssey CLx infrared scanner (Li-Cor- Nebraska USA). Relative protein levels were calculated by using internal reference β-actin.

### Gut Microbiota Analysis

About 10-mg fresh feces were obtained from each rat after a 15-day KD administration. The genome of gut microbiota was isolated using a FastDNA Spin Kit (Qbiogen, Carlsbad, CA, USA). 16S rRNA was amplified by PCR using forward primer, 5’-GAGAGTTTGATCCTGGCTCAG-3’ and the reverse primer, 5’-GGTTACCTTGTTACGACTT-3’. Gut microbiota was analyzed by using 16S rRNA sequencing. Heatmap and taxon relative abundance bar diagram was created by using custom R scripts and ggplot2.

### Statistical Analyses

Data are presented as the means ± standard error of the mean (SEM) and analyzed using the SPSS 21.0 software (SPSS, Inc., Chicago, IL, USA). Student’s t-test and one-way analysis of variance (ANOVA) with post-hoc Tukey’s tests were used to evaluate the variables between groups. Paeoniflorin and baicalin occupies the main proportion of KD and the Pearson correlation coefficient test was used to explore the relationship between serum levels and oxidative, and or inflammatory factors. The statistical difference was significant if the value of P <0.05.

## Results

### Paeoniflorin and Baicalin Were the Dominant Ingredients in KD

**Figure 1:**
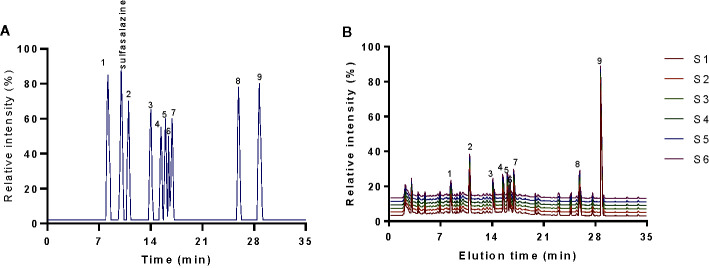
HPLC analysis of the main chemicals of kuijie granule. **(A)** standards. **(B)** the main chemicals of kuijie granule from 6 different batches. 1, gallic acid. 2, paeoniflorin. 3, emodin. 4, berberine. 5, coptisine. 6, palmatine. 7, jatrorrhizine. 8, baicalein. 9, baicalin.

HPLC analysis indicated that elution time of standards gallic acid (8.26 min), sulfasalazine (10.01 min), paeoniflorin (11.05 min), emodin (13.98 min), berberine (15.41 min), coptisine (15.96 min), palmatine (16.38 min), jatrorrhizine (16.87 min), baicalein (25.91 min) and baicalin (28.68 min) (**Figure 1A**), respectively. The main components of KD consist of gallic acid (12.6 ± 1.0 mg/g), paeoniflorin (21.2 ± 1.8 mg/g), emodin (13.0 ± 1.2 mg/g), berberine (14.3 ± 1.1 mg/g), coptisine (13.4 ± 0.8 mg/g), palmatine (14.3 ± 0.7 mg/g), jatrorrhizine (15.7 ± 1.0 mg/g), baicalein (14.1 ± 0.4 mg/g) and baicalin (126.6 ± 2.1 mg/g) (**Figure 1B**). The results imply that KD possibly displays its function *via* these important compounds.

**Figure 2:**
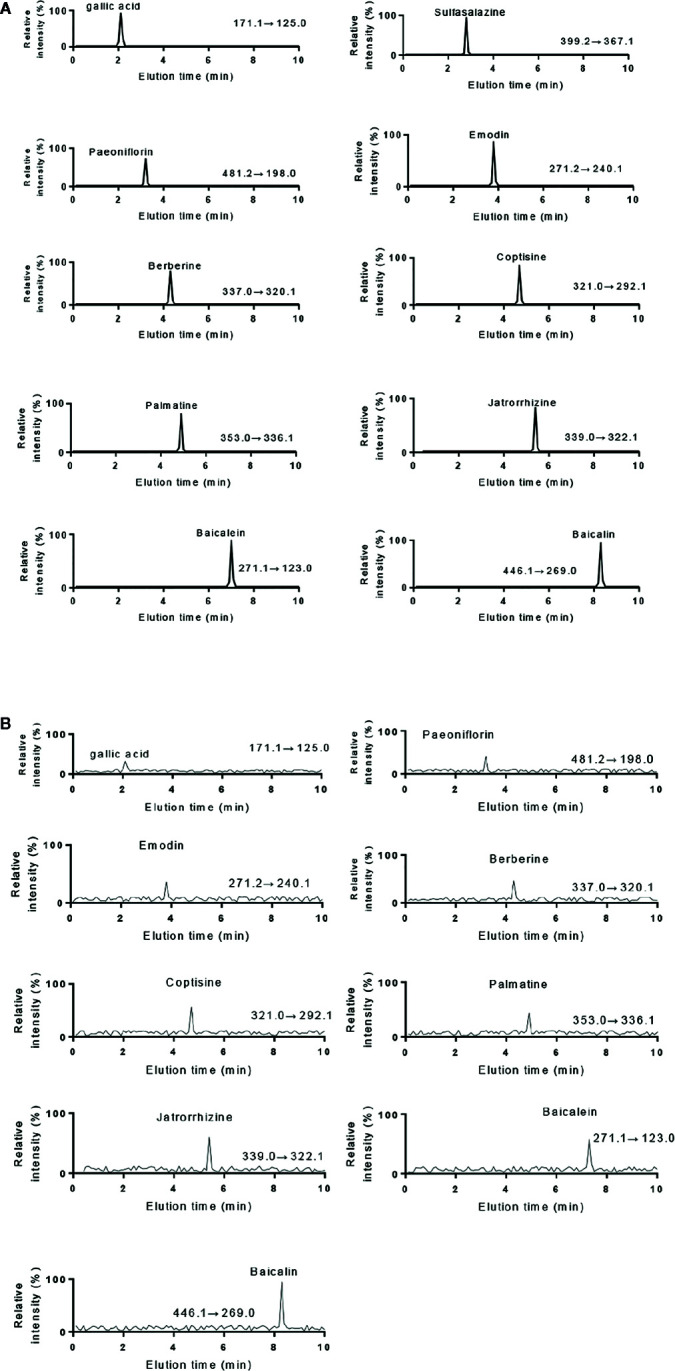
UPLC-MS/MS analysis of standards and KD extracts. **(A)** UPLC-MS/MS analysis of standards (gallic acid. sulfasalazine, paeoniflorin, emodin, berberine, coptisine, palmatine, jatrorrhizine, baicalein and baicalin). **(B)** UPLC-MS/MS analysis of KD extracts.

The HPLC results of standards and KD extracts were further confirmed by those from UPLC-MS/MS. UPLC-MS/MS analysis showed that elution time of standards gallic acid (2.12 min), sulfasalazine (2.81 min), paeoniflorin (3.24 min), emodin (3.82 min), berberine (4.31 min), coptisine (4.72 min), palmatine (4.93 min), jatrorrhizine (5.12 min), baicalein (7.03 min) and baicalin (8.25 min) (**Figure 2A**), respectively. The main components of KD consist of gallic acid, paeoniflorin, emodin, berberine, coptisine, palmatine, jatrorrhizine, baicalein and baicalin with the same elution time (**Figure 2B**).

### Serum Main Chemicals

**Figure 3:**
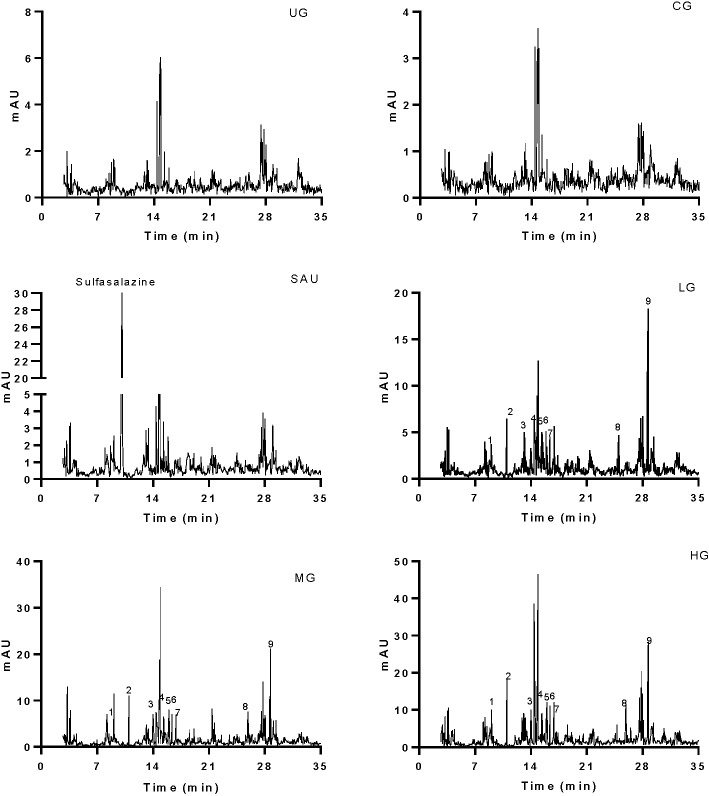
HPLC analysis of serum chemicals. The chemicals (sulfasalazine, paeoniflorin and baicalin) were determined according to the standards in **Figure 1A**. All rats were divided into seven groups, the vehicle (CG), UG (UG, UC model), sulfasalazine (SG, sulfasalazine treatment), low-dose KD (LG, 20 mg/kg daily), middle-dose KD (MG, 40 mg/kg) and high-dose KD (HG, 80 mg/kg) groups. 1, gallic acid. 2, paeoniflorin. 3, emodin. 4, berberine. 5, coptisine. 6, palmatine. 7, jatrorrhizine. 8, baicalein. 9, baicalin.

Serum main chemicals (gallic acid, paeoniflorin, emodin, berberine, coptisine, palmatine, jatrorrhizine, baicalein and baicalin) of KD were detected in the LG, MG and HG groups, respectively. Meanwhile, the proportion of these chemicals were increasing from the LG to the HG group (**Figure 3**). High-level of sulfasalazine (13.5 ± 1.8 µg/ml) was found in the SG group (**Figure 3**). No main chemical of KD and sulfasalazine were found in the serum from other groups (**Figure 3**).

### KD Treatment Increased Antioxidant Properties in the UC Model

**Figure 4:**
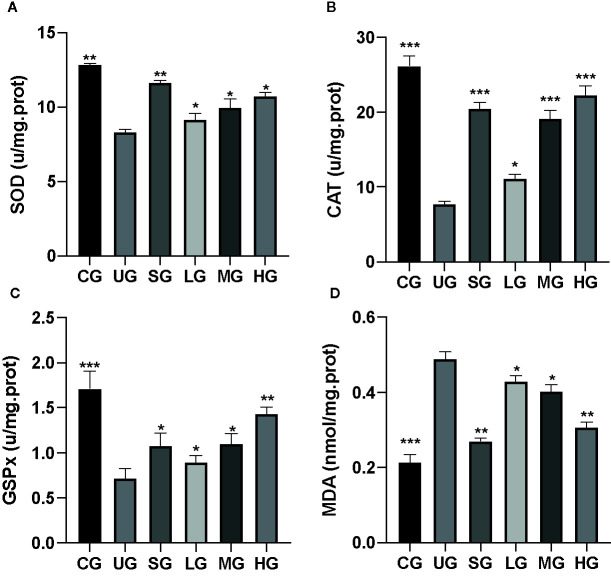
The effects of Kuijieyuan decoction on the levels of oxidative stress biomarkers. **(A)** SOD. **(B)** CAT. **(C)** GSPx. **(D)** MDA. *P < 0.05, **P < 0.01 and ***P < 0.001 vs the UG group. n = 8 for each group.

After the establishment of the UC model, the levels of SOD (**Figure 4A**), CAT (**Figure 4B**), and GSPx (**Figure 4C**) were decreased while MDA (**Figure 4D**) was increased in the UG group when compared with the CG group (P <0.05). Sulfasalazine and KD treatment increased the levels of SOD (**Figure 4A**), CAT (**Figure 4B**), and GSPx (**Figure 4C**) and reduced the level of MDA (**Figure 4D**, P <0.05). Especially, KD caused the changes of oxidative stress biomarkers in a dose-dependent way and the changes in the HG group were higher than those in the SG group (**Figure 4**, P <0.05).

### KD Treatment Increased Anti-Inflammatory Properties in the UC Model

**Figure 5:**
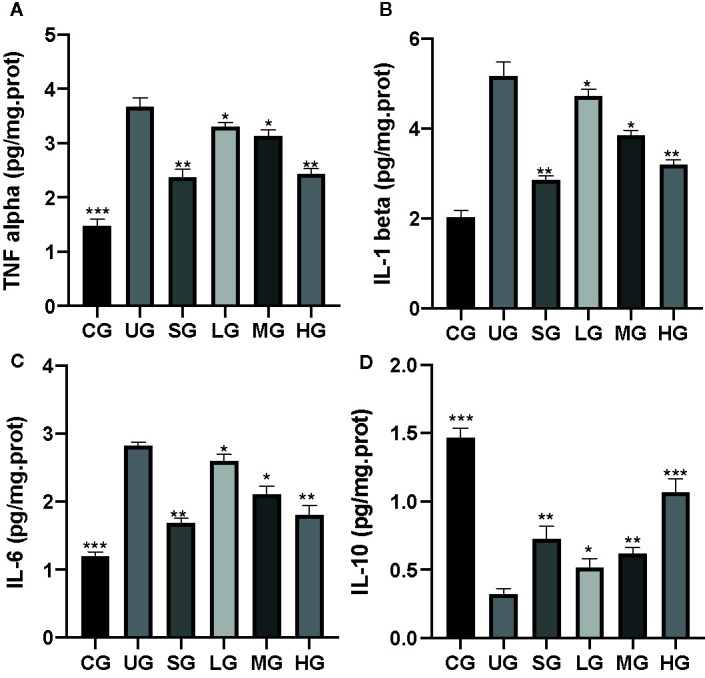
The effects of Kuijieyuan decoction on the levels of inflammatory cytokines. **(A)** TNF α. **(B)** IL-1β. **(C)** IL-6. **(D)** IL-10. *P < 0.05, **P < 0.01 and ***P < 0.001 vs the UG group. n = 8 for each group.

After the establishment of UC model, the levels of TNFα (**Figure 5A**), IL-1 (**Figure 5B**), and IL-6 (**Figure 5C**) were increased while the level of IL-10 (**Figure 5D**) was reduced in the UG group when compared with the CG group (P <0.05). Sulfasalazine and KD treatment reduced the level of TNFα (**Figure 5A**), IL-1 (**Figure 5B**), and IL-6 (**Figure 5C**) and increased the level of IL-10 (**Figure 5D**, P <0.05). Especially, KD caused the changes of inflammatory cytokines in a dose-dependent way and the IL-10 level in the HG group was increased more than that in the SG group (**Figure 5D**, P <0.05).

### KD Treatment Reduced DAI Scores and Increased Colon Length of UC Rat

**Figure 6:**
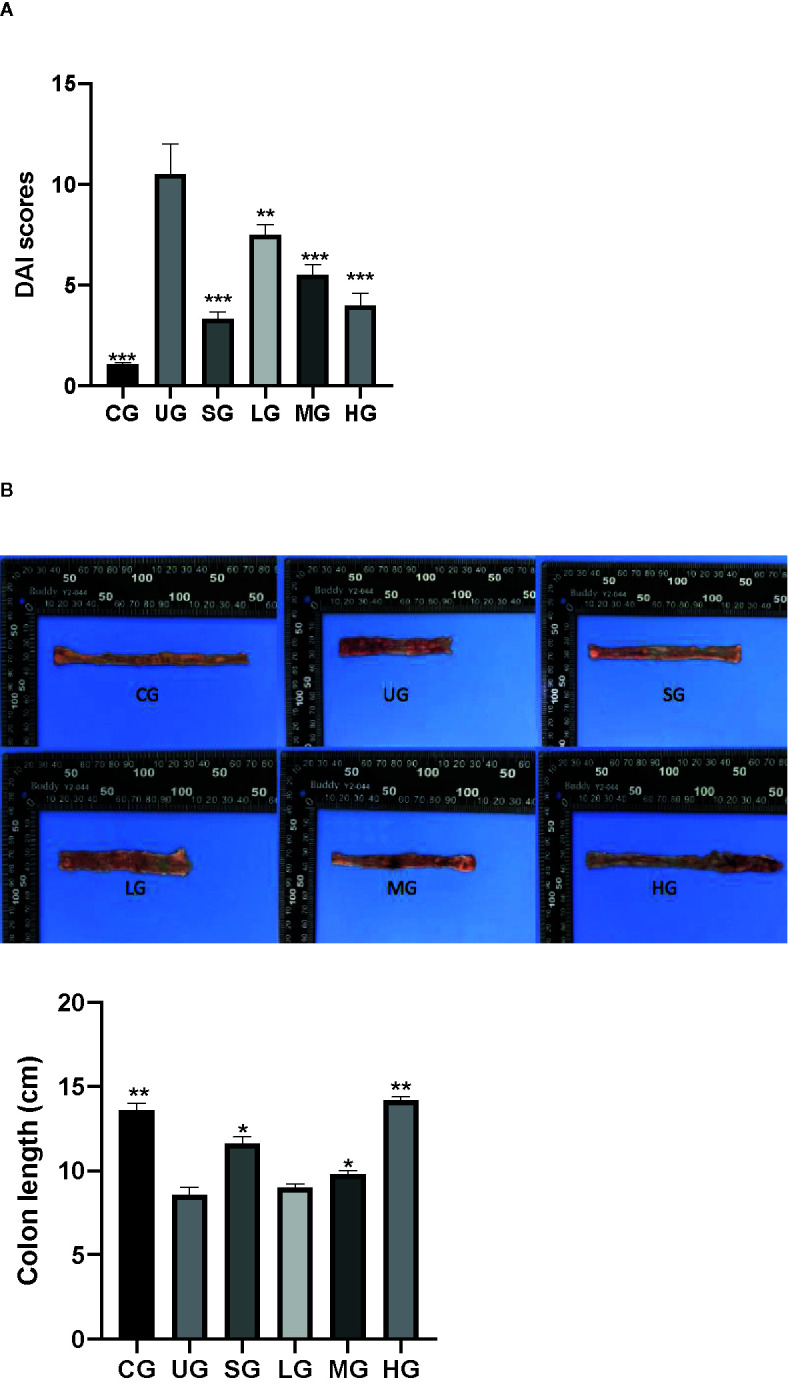
The effects of Kuijieyuan decoction on DAI scores and the colon length of UC rats. **(A)** DAI scores. **(B)** the colon length. n = 8 for each group. *P < 0.05, **P < 0.01 and ***P < 0.001.

After the establishment of the UC model, the DAI scores of UC rats were increased in the UG group when compared with those in the CG group (**Figure 6A**, P <0.05). Sulfasalazine and KD treatment reduced their DAI scores when compared with those in the UG group (**Figure 6A**, P <0.05). Especially, KD caused a reduction in the DAI scores in a dose-dependent way (**Figure 6A**, P <0.05).

After the establishment of UC model, the colon length of UC rats was reduced in the UG group when compared with that in the CG group (**Figure 6B**, P <0.05). Sulfasalazine and KD treatment increased their colon length when compared with that in the UG group (**Figure 6B**, P <0.05). Especially, KD caused the increase in the colon length in a dose-dependent way (**Figure 6B**, P <0.05).

### KD Treatment Improved Intestinal Barrier Injury of UC Rat

**Figure 7:**
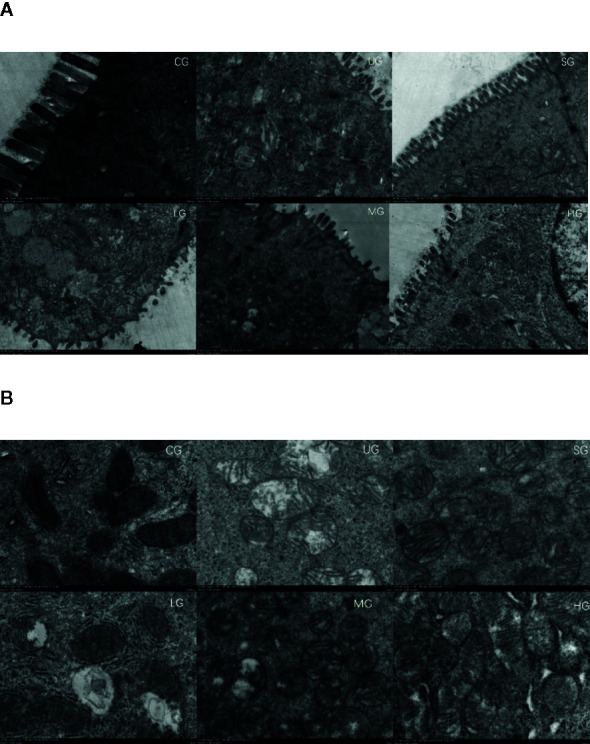
Scanning electron microscopy (SEM) observation of the intestinal barrier among different groups. **(A)** intestinal villi. **(B)** the structure of intestinal mitochondria. n = 8 for each group.

SEM analysis showed that after the establishment of UC model, the intestinal barriers of UC rats were damaged with reduced amounts of intestinal villi in the UG group when compared with those in the CG group (**Figure 7A**, P <0.05). Sulfasalazine and KD treatment increased the amounts of intestinal villi when compared with those in the UG group (**Figure 7A**, P <0.05). Especially, KD caused an increase in the amounts of intestinal villi in a dose-dependent way (**Figure 7A**, P <0.05). After the establishment of UC model, the structure of intestinal mitochondria in UC rats was damaged with the reduced number of ridges in the UG group when compared with that in the CG group (**Figure 7B**, P <0.05). Sulfasalazine and KD treatment increased the amounts of mitochondrial ridges and repaired the mitochondrial structure when compared with those in the UG group (**Figure 7B**, P <0.05). Especially, KD caused an increase in the number of mitochondrial ridges in a dose-dependent way (**Figure 7B**, P <0.05).

### KD Treatment Reduced Pathological Scores of UC

**Figure 8:**
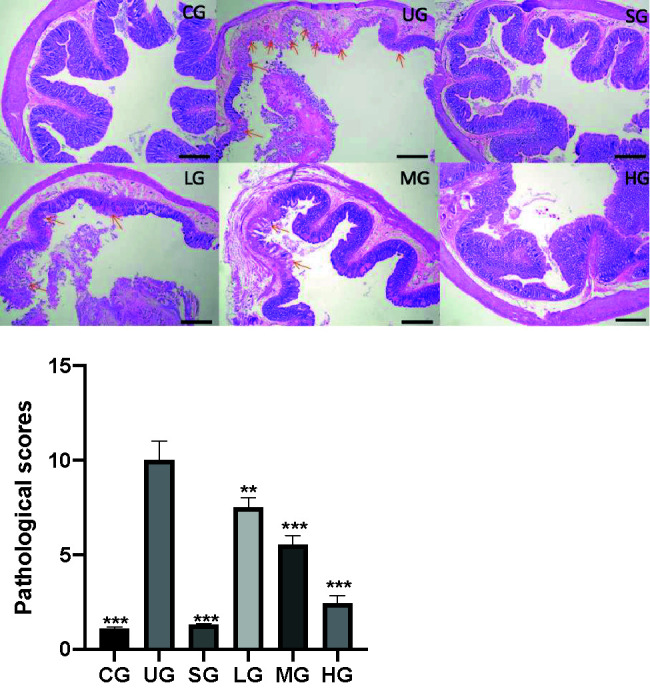
The effects of Kuijieyuan decoction on pathological scores of pancreas tissues. The colon mucosa one or more layers of epithelial cells were destroyed as arrow indicated. **P < 0.01 and ***P < 0.001 vs the UG group. n = 8 for each group.

After the establishment of the UC model, the infiltration of the colon by immature neoplastic myeloid (**Figure 8**) and pathological scores (**Figure 8**) were increased in the UG group when compared with those in the CG group (P <0.05). Sulfasalazine and KD treatment reduced the infiltration of pancreas by immature neoplastic myeloid and pathological scores (**Figure 8**, P <0.05). Especially, KD caused a reduction in the pathological scores in a dose-dependent way (**Figure 8**, P <0.05).

### KD Treatment Reduced TLR-Dependent PI3K/AKT/NF-κB Signaling in the UC Model

**Figure 9:**
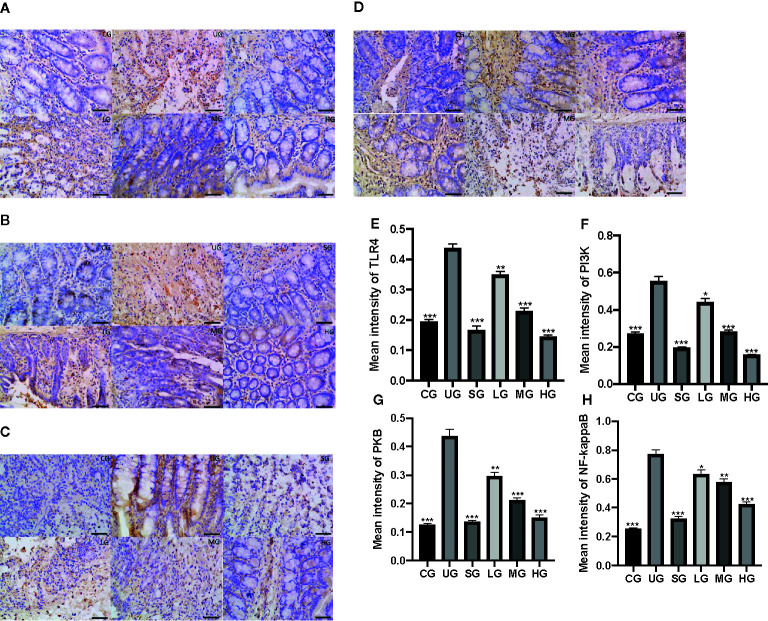
The effects of Kuijieyuan decoction on the expression of TLR4-dependent PI3K/AKT/NF-κB. **(A)** immunohistochemical analysis of TLR4 in the colon tissues. **(B)** immunohistochemical analysis of p-P3IK in the colon tissues. **(C)** immunohistochemical analysis of p-AKT in the colon tissues. **(D)** immunohistochemical analysis of p-NF-κB in the pancreas. **(E)** quantification of TLR4 levels in five different immunohistochemical images. **(F)** quantification of p-P3IK levels in five different immunohistochemical images. **(G)** quantification of p-AKT levels in five different immunohistochemical images. **(H)** quantification of p-NF-κB levels in five different immunohistochemical images. *P < 0.05, **P < 0.01 and ***P < 0.001 vs the UG group. n = 8 for each group.

IHC analysis showed that the brown staining of TLR4 was increased in the UG group when compared with that in the CG group (**Figure 9A**, P <0.05). Sulfasalazine and KD treatment reduced the brown staining and KD caused the changes in a dose-dependent way (**Figure 9A**, P <0.05). Similarly, the brown staining of p-P3IK was increased in the UG group when compared with that in the CG group (**Figure 9B**, P <0.05). Sulfasalazine and KD treatment reduced the brown staining and KD caused the changes in a dose-dependent way (**Figure 9B**, P <0.05). In contrast, the brown staining of p-AKT increased in the UG group when compared with that in the CG group (**Figure 9C**, P <0.05). Sulfasalazine and KD treatment reduced the brown staining and KD caused the changes in a dose-dependent way (**Figure 9C**, P <0.05). The brown staining of p-NF-κB was increased in the UG group when compared with that in the CG group (**Figure 9D**, P <0.05). Sulfasalazine and KD treatment reduced the brown staining and KD caused the changes in a dose-dependent way (**Figure 9D**, P <0.05). Quantity analysis also showed that the UC model establishment reduced the protein levels of TLR4 (**Figure 9E**), p-P3IK (**Figure 9F**), p-AKT (**Figure 9G**) and p-NF-κB (**Figure 9H**, P <0.05). Sulfasalazine and KD treatment reduced the protein levels of TLR4 (**Figure 9E**), p-P3IK (**Figure 9F**), p-AKT (**Figure 9G**) and p-NF-κB (**Figure 9H**) in a dose-dependent way (P <0.05).

### KD Treatment Affected Relative mRNA Levels of TLR4/PI3K/AKT/NF-κB in the UC Model

**Figure 10:**
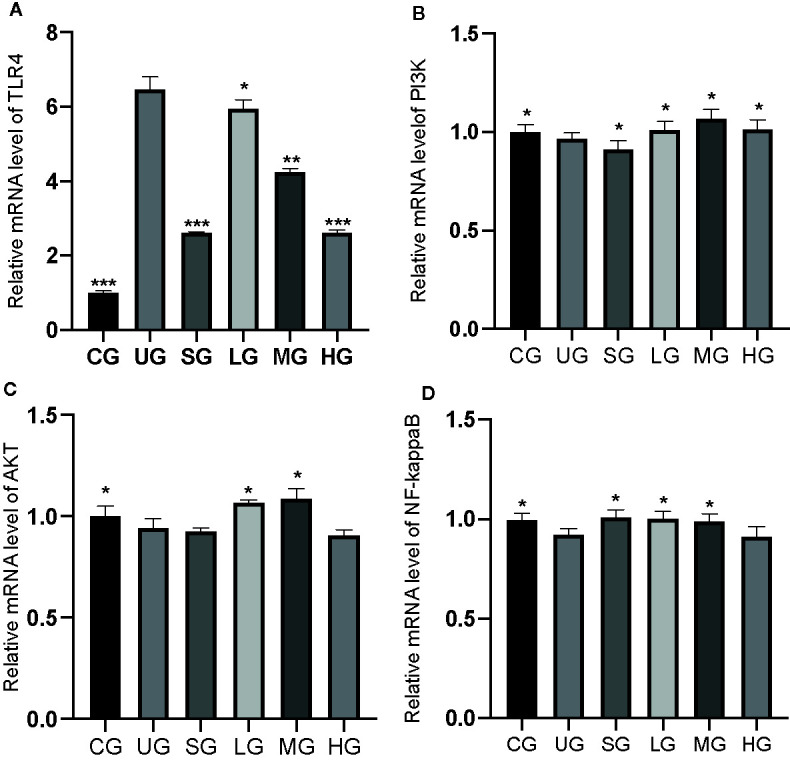
The effects of Kuijieyuan decoction on the relative mRNA levels of TLR4-dependent PI3K/AKT/NF-κB. **(A)** TLR4. **(B)** P3IK. **(C)** AKT. **(D)** NF-κB. *P < 0.05, **P < 0.01 and ***P < 0.001 vs the UG group. n = 8 for each group.

The RT-qPCR analysis showed that the relative mRNA levels of TLR4 was increased in the UG group when compared with those in the CG group (**Figure 10A**, P <0.05). Sulfasalazine and KD treatment reduced the levels and KD caused the changes in a dose-dependent way (**Figure 10A**, P <0.05). Similarly, relative mRNA level of P3IK was reduced in the UG group when compared with that in the CG group (**Figure 10B**, P <0.05). Sulfasalazine treatment reduced the level further while KD treatment increased the level (**Figure 10B**, P <0.05). In contrast, relative mRNA level of AKT was reduced in the UG group when compared with that in the CG group (**Figure 10C**, P <0.05). Sulfasalazine and high-dose KD did not change the level and low-dose and middle-dose KD treatment increased the level (**Figure 10C**, P <0.05). Relative mRNA level of NF-κB was reduced in the UG and HG groups while sulfasalazine, low-dose and middle-dose KD treatment increased the level of NF-κB (**Figure 10D**, P <0.05). KD treatment affected relative mRNA levels of TLR4/PI3K/AKT/NF-κB in the UC model.

### KD Treatment Reduced Relative Protein Levels of TLR-Dependent Phosphorylated PI3K/AKT/NF-κB in the UC Model

**Figure 11:**
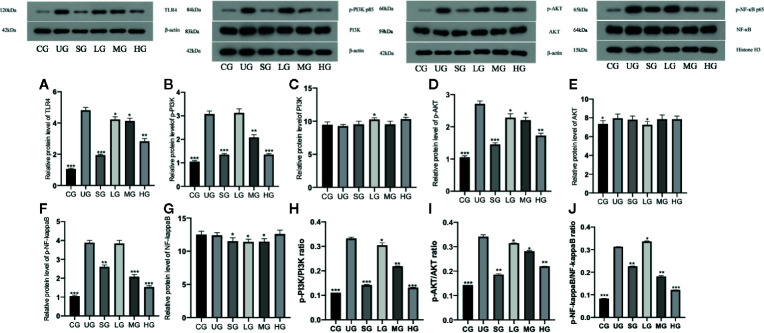
The effects of Kuijieyuan decoction on the relative protein levels of TLR4-dependent phosphorylated PI3K/AKT/NF-κB. **(A)** TLR4. **(B)** p-P3IK. **(C)** P3IK. **(D)** p-AKT. **(E)** AKT. **(F)** p-NF-κB. **(G)** NF-κB. **(H)** the ratios of p-P3IK/P3IK. **(I)** the ratio of p-AKT/AKT. **(J)** the ratio of p-NF-κB/NF-κB. *P < 0.05, **P < 0.01 and ***P < 0.001 vs the UG group. n = 8 for each group.

Western Blot analysis showed that relative protein level of TLR4 was increased in the UG group when compared with the CG group (**Figure 11A**, P <0.05). Sulfasalazine and KD treatment reduced the level of TLR4 and KD caused the changes in a dose-dependent way (**Figure 11A**, P <0.05). Similarly, relative protein level of p-P3IK was increased in the UG group when compared with that in the CG group (**Figure 11B**, P <0.05). Sulfasalazine and KD treatment reduced the level and KD caused the changes in a dose-dependent way (**Figure 11B**, P <0.05) while relative protein level of P3IK was less significant among all groups (**Figure 11C**). In contrast, relative protein level of p-AKT increased in the UG group when compared with that in the CG group (**Figure 11D**, P <0.05). Sulfasalazine and KD treatment reduced the level of p-AKT and KD caused the changes in a dose-dependent way (**Figure 11D**, P <0.05) while relative protein level of AKT was less significant among all groups (**Figure 11E**). Relative protein level of p-NF-κB was increased in the UG group when compared with that in the CG group (**Figure 11F**, P <0.05). Sulfasalazine and KD treatment reduced the level of p-NF-κB and KD caused the changes in a dose-dependent way (**Figure 11F**, P <0.05) while relative protein level of NF-κB was less significant among all groups (**Figure 11G**). The results suggest that KD treatment reduces relative protein levels of TLR-dependent phosphorylated PI3K/AKT/NF-κB in the UC model. On the other hand, the changing trend for the ratios of p-P3IK/P3IK (**Figure 11H**), p-AKT/AKT (**Figure 11I**) and p-NF-κB/NF-κB (**Figure 11J**) was similar with the changing trend of phosphorylating status of p-P3IK (**Figure 11B**), p-AKT (**Figure 11E**) and p-NF-κB (**Figure 11G**).

### The Serum Levels of Paeoniflorin and Baicalin Had a Strong Correlation With the Levels of Inflammatory and Oxidative Stress Biomarkers

**Figure 12:**
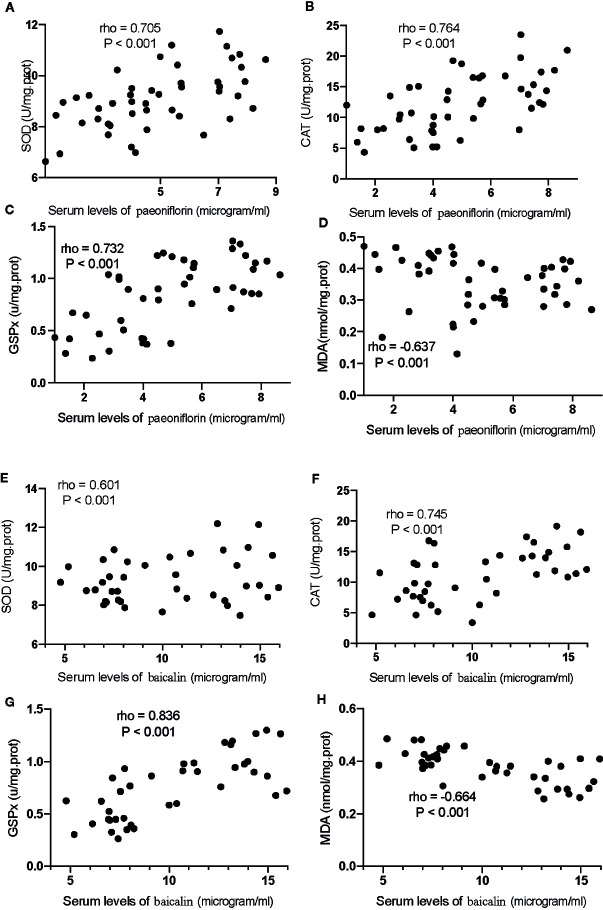
The strong correlation between the levels of oxidative stress biomarkers and paeoniflorin or baicalin. **(A)** SOD and paeoniflorin. **(B)** CAT and paeoniflorin. **(C)** GSPx and paeoniflorin. **(D)** MDA and paeoniflorin. **(E)** SOD and baicalin. **(F)** CAT and baicalin. **(G)** GSPx and baicalin. **(H)** MDA and baicalin. There was a strong positive correlation if rho >0.5 and or a strong negative correlation if rho <−0.5.

For oxidative stress biomarkers, the increase in the serum levels of paeoniflorin would cause the increase in the levels of SOD (**Figure 12A**), CAT (**Figure 12B**) and GSPx (**Figure 12C**), and the reduction in the levels of MDA (**Figure 12D**, P <0.001). Similarly, the increase in the serum levels of baicalin would cause an increase in the levels of SOD (**Figure 12E**), CAT (**Figure 12F**) and GSPx (**Figure 12G**), and the reduction in the levels of MDA (**Figure 12H**, P <0.001). All these results suggest that the levels of paeoniflorin and baicalin had a strong correlation with the levels of inflammatory and oxidative stress biomarkers.

**Figure 13:**
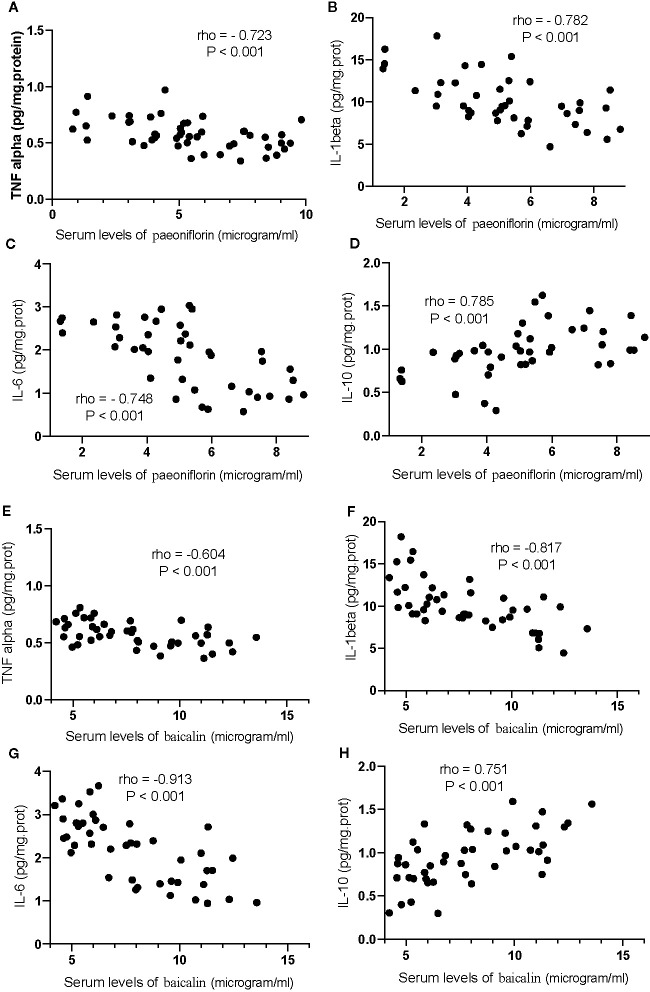
The strong correlation between the levels of inflammatory cytokines and paeoniflorin or baicalin. **(A)** TNFα and paeoniflorin. **(B)** IL-1 β and paeoniflorin. **(C)** IL-6 and paeoniflorin. **(D)** IL-10 and paeoniflorin. **(E)** TNFα and baicalin. **(F)** IL-1 β and baicalin. **(G)** IL-6 and baicalin. **(H)** IL-10 and baicalin. There was a strong positive correlation if rho >0.5 and or a strong negative correlation if rho <−0.5.

Correlation test showed that an increase in the serum levels of paeoniflorin would cause the reduction in the levels of TNFα (**Figure 13A**), IL-1β (**Figure 13B**) and IL-6 (**Figure 13C**), and the increase in the levels of IL-10 (**Figure 13D**, P <0.001). Similarly, the increase in the serum levels of baicalin would cause a reduction in the levels of TNFα (**Figure 13E**), IL-1β (**Figure 13F**) and IL-6 (**Figure 13G**), and the increase in the levels of IL-10 (**Figure 13H**, P <0.001).

### KD Treatment Improved Gut Microbiota in the UC Model

**Figure 14:**
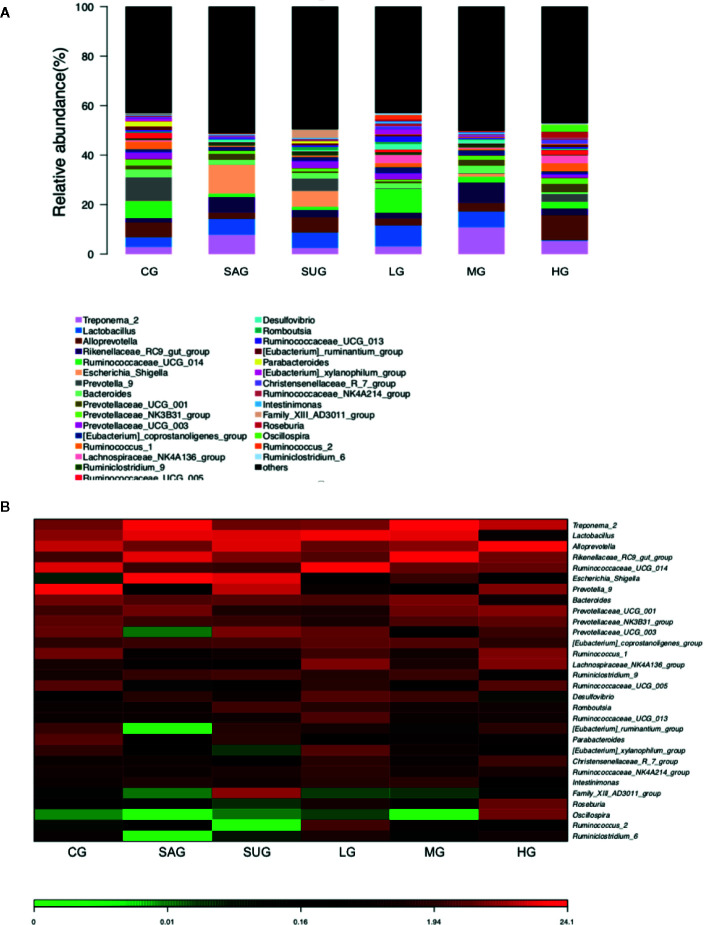
The composition of gut microbiota among different groups. **(A)** the proportion of gut microbiota. **(B)** heatmap analysis of gut microbiota changes from different treatments.

Bar plot showed that the proportion of Alloprevotella, Treponema, Prevotellaceae, and Prevotella was decreased and the levels of Escherichia_Shigella and Desulfovibrio were increased in the UG group when compared with the CG group (**Figure 14A**). KD treatment increased the proportion of Alloprevotella, Treponema, Prevotellaceae, and Prevotella and decreased the proportion of Escherichia_Shigella and Desulfovibrio in the UC model (**Figure 14A**). Heatmap also showed that the proportion of Alloprevotella, Treponema, Prevotellaceae, and Prevotella were decreased and the proportion of Escherichia_Shigella and Desulfovibrio were increased in the SG group when compared with the CG group (**Figure 14B**). KD treatment increased the proportion of Alloprevotella, Treponema, Prevotellaceae, and Prevotella and decreased the proportion of Escherichia_Shigella and Desulfovibrio in the model (**Figure 14B**). The results suggest that KD treatment improved gut microbiota in the UC model.

## Discussion

In the present experiment, the administration of DSS was conducted to establish the UC model, DAI scores and colon length (**Figure 6**), and pathological changes (**Figure 8**) were found in the UG (UC model) group when compared with those in the CG group. The results suggest that the DSS induced UC symptoms in rats, which had high DAI scores, short colon (**Figure 6**), and higher pathological scores in the tissues (**Figure 8**). Meanwhile, the inflammatory responses and oxidative stress were also increased (**Figures 3** and **4**), suggesting that DSS induction is an effective method to create a UC model and also used in other UC studies (Wan et al., 2018; Hou et al., 2019; Ye et al., 2019).

KD treatment repaired the intestinal barrier injury caused by the UC model (**Figure 7**) and improved the gut microbiota in the model (**Figure 14**). The improvement of intestinal barrier injury and gut microbiota will reduce bacterial infection, and oxidative and inflammatory responses. These results may contribute to an increase in antioxidant and anti-inflammatory abilities. The improvement of antioxidant and anti-inflammatory properties is the potential approaches in the prevention of UC progression (Shalkami et al., 2018; Stan et al., 2019). In the present work, KD protected rats against UC development by increasing antioxidant and anti-inflammatory capacities (**Figures 3** and **4**). HPLC analysis showed that there were nine compounds in the main ingredients of KD while paeoniflorin and baicalin occupied the more proportion when compared other compounds (**Figure 1B**). On the other hand, the serum levels of these compounds were increased with the increase in the oral administration of KD (**Figure 3**). The serum levels of paeoniflorin and baicalin had a strong correlation with the levels of inflammatory and oxidative stress biomarkers (**Figures 11** and **12**). All these results demonstrated that KD possibly increase the antioxidant and anti-inflammatory activities in the UC models through its main ingredients.

KD treatment also affected the expression levels of TLR-dependent phosphorylated PI3K/AKT/NF-κB (**Figures 8** and **10**). The present findings were consistent with previous reports that UC induction will increase the levels of TLR-dependent phosphorylated PI3K/AKT/NF-κB (Zhang et al., 2015; Zhao et al., 2020). According to a previous report, the PI3K-Akt pathway was activated through their phosphorylation (Guha and Mackman, 2002). Transcription factor NF-κB plays a central role in the induction of various inflammatory signals while the phosphorylation of NF-κB p65 subunit has been implicated as a key step to promote target gene expression by assisting promoter transition from an inactive state to an active state (Vermeulen et al., 2003). Therefore, KD treatment activated TLR-dependent phosphorylated PI3K/AKT/NF-κB signaling pathway by promoted their phosphorylation.

KD treatment reduced UC injuries and increased serum levels of paeoniflorin and baicalin (**Figure 3**). The result was consistent with a previous report that paeoniflorin had the anti-inflammatory properties in UC by inactivating MAPK/NF-kappaB pathway and apoptosis in the rat model (Gu et al., 2017). Baicalin also had inhibitory effects on UC risk by inhibiting IKK/IKB/NF-kB signaling pathway and apoptosis-related proteins in a UC model (Shen et al., 2019). On the other hand, sulfasalazine and KD had a similar function in the prevention of UG progression, suggestion KD may be a potential drug to replace sulfasalazine with fewer side effects as natural products.

KD treatment increased the proportion of Alloprevotella, Treponema, Prevotellaceae, and Prevotella and reduced the proportion of Escherichia_Shigella and Desulfovibrio in the high-dose group (**Figure 14**). The results suggest that KD selectively promoted the growth of Alloprevotella, Treponema, Prevotellaceae, and Prevotella, and inhibited Escherichia_Shigella and Desulfovibrio. Perilla oil (rich in the omega-3 fatty acids with high-level antioxidant activity) treatment also facilitated the richness of Alloprevotella in the intestine and restored gut microflora diversity (Wang et al., 2020). Treponema and Prevotellaceae was reported to be significantly increased in the lysozyme-treated groups, and the microbial genes related to glycerolipid, propanoate, and pyruvate metabolism were highly expressed to promote the enrichment of probiotics in the gut microbiota (Gomez et al., 2016). In contrast, intestinal inflammation is associated with an increase in Escherichia/Shigella (Kostic et al., 2014) and Desulfovibrio (Inness et al., 2007). These results suggest that KD treatment improved gut microbiota by increasing beneficial bacteria and reducing harmful bacteria. Notably, the establishment of UC model decreased the proportion of Alloprevotella, Treponema, Prevotellaceae, and Prevotella and increased the levels of Escherichia_Shigella and Desulfovibrio (**Figure 14A**). The results were not consistent with those in the previous work that DSS treatment decreased the probiotics Lactobacillus, Roseburia and Pectobacterium, and increased Firmicutes/Bacteroidetes ratio (Ma et al., 2018). The difference may be caused by the model establishment at different times. Furthermore, 3% DSS (w/v) was still given to the rats to avoid self-cure during the administration of different agents in the latter work.

There were some limitations in the present study. The HPLC results indicated that paeoniflorin and baicalin occupied the main proportion of nine compounds in KD. Paeoniflorin shows protective effects on UC models by inhibiting NF-kB pathway and apoptosis process (Gu et al., 2017). Bicalin has been reported to regulate IKK/IKB/NF-kB pathway and apoptotic proteins in the UC model (Shen et al., 2019). The anti-inflammatory effect is also found in the combination of paeoniflorin and baicalin in oral inflammatory diseases (Yu et al., 2019). For other compounds, berberine and gallic acid have been found to show anti-inflammatory function in atopic dermatitis-like skin inflammation (Tsang et al., 2016). Emodin can ameliorate bleomycin-induced pulmonary fibrosis through anti-inflammatory and anti-oxidative properties (Tian et al., 2018). Coptisine exerts anti-inflammatory activity by inhibiting NF-κB and MAPK signaling pathways in an animal model (Chen et al., 2017). Palmatine displays anti-inflammatory function by inhibiting TRIF-dependent NF-κB (Yan et al., 2017). Jatrorrhizine suppresses proliferation, migration, and secretion of synoviocytes in an animal model with rheumatoid arthritis (Qiu et al., 2018). Baicalein exerts anti-inflammatory function by inhibiting NF-κB transactivation (Patwardhan et al., 2016). All these compounds may show multi-anti-inflammatory mechanisms in the therapy of intestinal barrier injury of the UC model. However, the effects of the combination of the nine compounds should be tested in the UC model in the future.

## Data Availability Statement

The datasets analyzed in this article are not publicly available. Requests to access the datasets should be directed to XP, piaoxuehua1982@126.com.

## Ethics Statement

The animal study was reviewed and approved by The First affiliated Hospital, Jinzhou Medical University.

## Author Contributions

BL, XP, WN, QZ, CM, TW and QG designed and performed the whole experiment, and analyzed all data. TC and SL analyzed all data and wrote the paper. All authors contributed to the article and approved the submitted version.

## Conflict of Interest

The authors declare that the research was conducted in the absence of any commercial or financial relationships that could be construed as a potential conflict of interest.
