# Supplementary material for: Kuijieyuan Decoction Improved Intestinal Barrier Injury of Ulcerative Colitis by Affecting TLR4-Dependent PI3K/AKT/NF-κB Oxidative and Inflammatory Signaling and Gut Microbiota
Source: Front Pharmacol. 2020 Jul 29;11:1036. doi: 10.3389/fphar.2020.01036 (PMC7403404; doi:10.3389/fphar.2020.01036)
Supplement: Supplementary file 1 [file DataSheet_1.doc]

Kuijieyuan decoction (KD) was purchased from Tianjin Tasly Pharmaceutical Co., Ltd. (batch number 60060101, Tianjin, China) and prepared as follows (Voucher number): 30 grams of *Astragalus mongholicus Bunge* (20100478), 40 grams of *Hedyotis diffusa Willd* (PS1034MT01), 10 grams of *Cirsium undulatumn (Nutt.) Spreng*, 10 grams of *Cirsium setosum (Willd.) M. Bieb* (PS0611MT04), 40 grams of *Pulsatilla vulgaris Mill*, 10 grams of *Prunella vulgaris L. subsp. vulgaris* (MIB:ZPL:03560), 10 grams of *Coptis chinensis Franch*(PS0915MT01), 15 grams of *Polygonum cuspidatum Siebold & Zucc.*, 15 grams of *Atractylodes lancea (Thunb) DC* (SKP 051011201) and 10 grams of *Glycyrrhiza glabra L.*) (8600100). The herbs were rinsed and soaked in 2-liter cold water for 30 min, then boiled in water under reflux for 2 h. After concentration and filtration, the medicinal solution was concentrated to 1.0 g/ml containing crude drugs, and stored in a refrigerator at 4 ° C.

*Astragalus mongholicus Bunge*

a. The name of the person who authenticated the samples

Professor Liu He from Changchun University of Traditional Chinese Medicine

b. Authentication documents

Please refer to supporting files

c. Where the samples are kept for posterity and where they can be accessed from

Tianjin Tasly Pharmaceutical Co., Ltd. (batch number 60060101, Tianjin, China)

d. The botanical scans


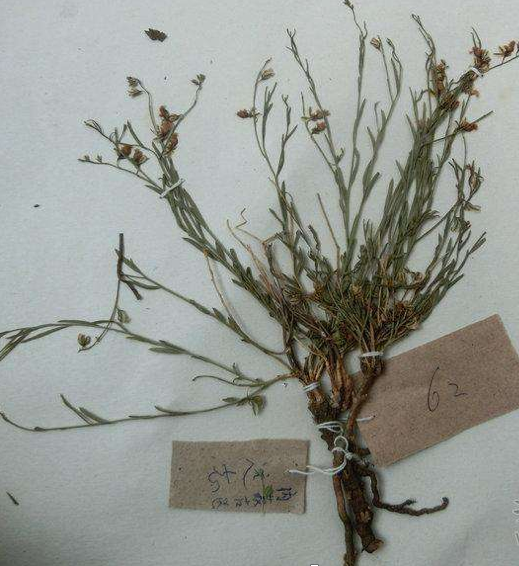


e. The coordinates of plant picking

42 °-45 ° north latitude, 108 ° -114 ° east longitude

*Hedyotis diffusa*

a. The name of the person who authenticated the samples

Professor Liu He from Changchun University of Traditional Chinese Medicine

b. Authentication documents

Please refer to supporting files

c. Where the samples are kept for posterity and where they can be accessed from

Tianjin Tasly Pharmaceutical Co., Ltd. (batch number 60060101, Tianjin, China)

d. The botanical scans


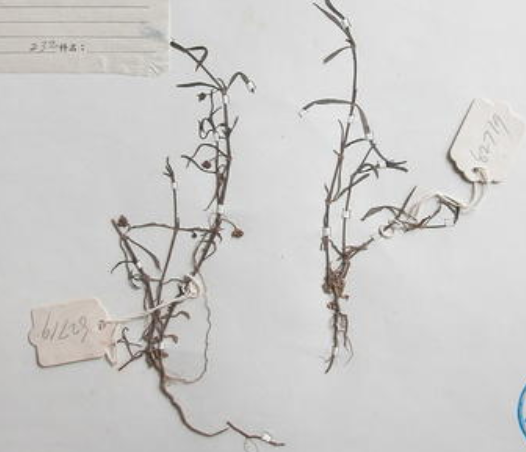


e. The coordinates of plant picking

28°16′36″—28°29′46″ north latitude, 116°31′17″—116°40′6″east longitude

*Cirsium undulatumn (Nutt.) Spreng*

a. The name of the person who authenticated the samples

Professor Liu He from Changchun University of Traditional Chinese Medicine

b. Authentication documents

Please refer to supporting files

c. Where the samples are kept for posterity and where they can be accessed from

Tianjin Tasly Pharmaceutical Co., Ltd. (batch number 60060101, Tianjin, China)

d. The botanical scans


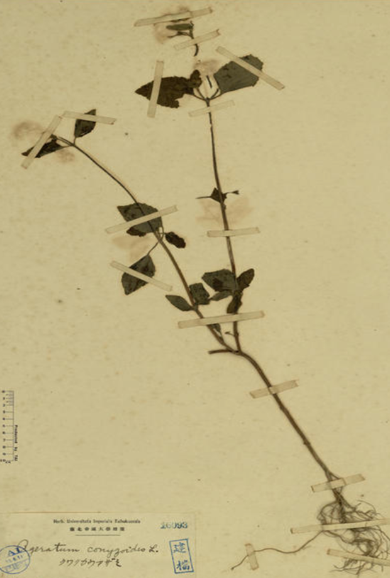


e. The coordinates of plant picking

119°26′45″ —120°28′17″ north latitude, 32°01′25″—32°28′16″east longitude

*Cirsium setosum*

a. The name of the person who authenticated the samples

Professor Liu He from Changchun University of Traditional Chinese Medicine

b. Authentication documents

Please refer to supporting files

c. Where the samples are kept for posterity and where they can be accessed from

Tianjin Tasly Pharmaceutical Co., Ltd. (batch number 60060101, Tianjin, China)

d. The botanical scans


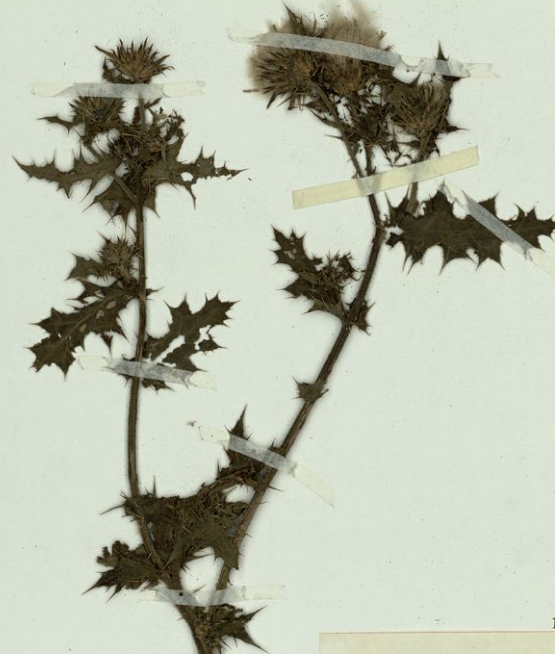


e. The coordinates of plant picking

30°2′-30°9′north latitude, 110°29′-110°41′ east longitude

*Pulsatilla vulgaris Mill*

a. The name of the person who authenticated the samples

Professor Liu He from Changchun University of Traditional Chinese Medicine

b. Authentication documents

Please refer to supporting files

c. Where the samples are kept for posterity and where they can be accessed from

Tianjin Tasly Pharmaceutical Co., Ltd. (batch number 60060101, Tianjin, China)

d. The botanical scans


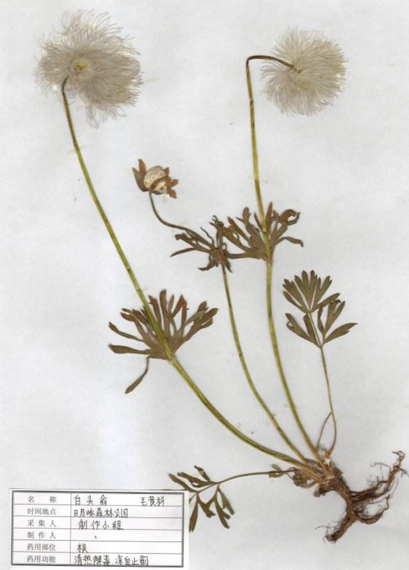


e. The coordinates of plant picking

42°06′～42°48′ north latitude, 126°30′～127°16′ east longitude

*Prunella vulgaris L. subsp. Vulgaris*

a. The name of the person who authenticated the samples

Professor Liu He from Changchun University of Traditional Chinese Medicine

b. Authentication documents

Please refer to supporting files

c. Where the samples are kept for posterity and where they can be accessed from

Tianjin Tasly Pharmaceutical Co., Ltd. (batch number 60060101, Tianjin, China)

d. The botanical scans


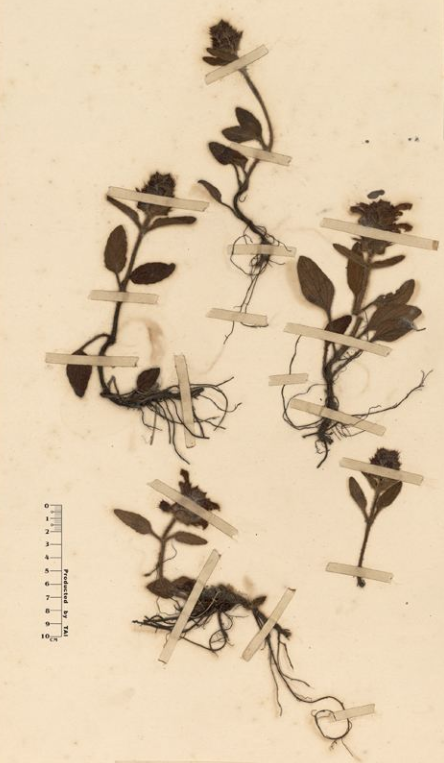


e. The coordinates of plant picking

32°17′～32°43′ north latitude, 113°00′～113°49′ east longitude

*Coptis chinensis Franch*

a. The name of the person who authenticated the samples

Professor Liu He from Changchun University of Traditional Chinese Medicine

b. Authentication documents

Please refer to supporting files

c. Where the samples are kept for posterity and where they can be accessed from

Tianjin Tasly Pharmaceutical Co., Ltd. (batch number 60060101, Tianjin, China)

d. The botanical scans


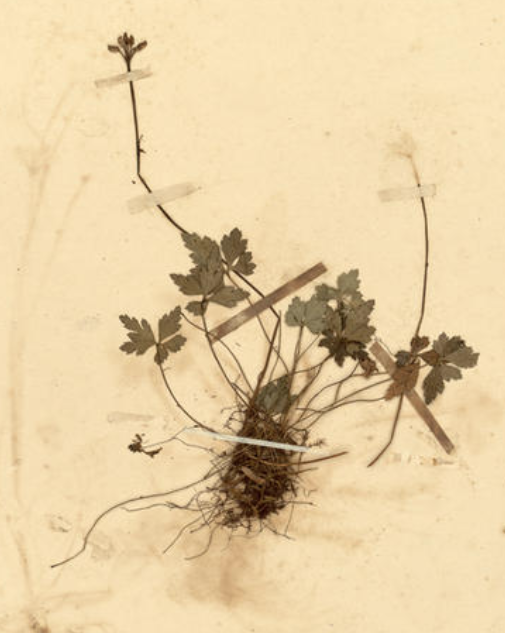


e. The coordinates of plant picking

22°~33° north latitude, 97°~122° east longitude

*Polygonum cuspidatum Siebold & Zucc.*

a. The name of the person who authenticated the samples

Professor Liu He from Changchun University of Traditional Chinese Medicine

b. Authentication documents

Please refer to supporting files

c. Where the samples are kept for posterity and where they can be accessed from

Tianjin Tasly Pharmaceutical Co., Ltd. (batch number 60060101, Tianjin, China)

d. The botanical scans


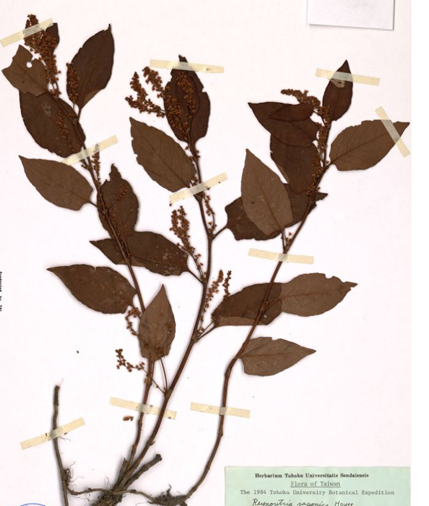


e. The coordinates of plant picking

31°34′～ 32°31′north latitude, 110°02′～111°15′east longitude

*Atractylodes lancea (Thunb) DC*

a. The name of the person who authenticated the samples

Professor Liu He from Changchun University of Traditional Chinese Medicine

b. Authentication documents

Please refer to supporting files

c. Where the samples are kept for posterity and where they can be accessed from

Tianjin Tasly Pharmaceutical Co., Ltd. (batch number 60060101, Tianjin, China)

d. The botanical scans


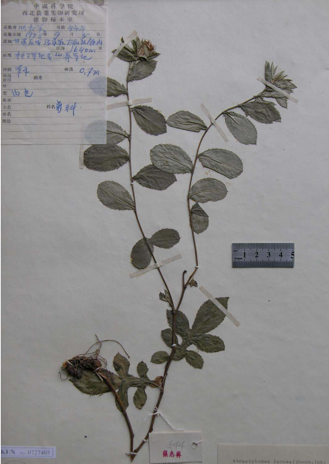


e. The coordinates of plant picking

31.7° north latitude,121° east longitude

*Glycyrrhiza glabra L.*

a. The name of the person who authenticated the samples

Professor Liu He from Changchun University of Traditional Chinese Medicine

b. Authentication documents

Please refer to supporting files

c. Where the samples are kept for posterity and where they can be accessed from

Tianjin Tasly Pharmaceutical Co., Ltd. (batch number 60060101, Tianjin, China)

d. The botanical scans


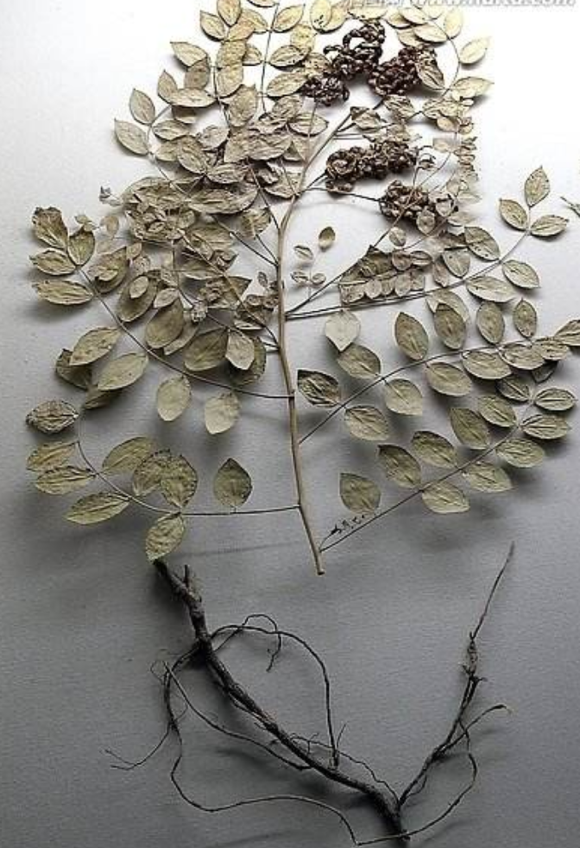


e. The coordinates of plant picking

37°～50° north latitude, 75°～123° east longitude
